# Processing-Induced Markers in Proteins of Commercial Plant-Based Drinks in Relation to Compositional Aspects

**DOI:** 10.3390/foods12173282

**Published:** 2023-09-01

**Authors:** Ida Schwartz Roland, Miguel Aguilera-Toro, Søren Drud-Heydary Nielsen, Nina Aagaard Poulsen, Lotte Bach Larsen

**Affiliations:** Department of Food Science, Aarhus University, Agro Food Park 48, DK-8200 Aarhus, Denmark; isr@food.au.dk (I.S.R.);

**Keywords:** plant-based drinks, Maillard reaction, processing, furosine, lanthionine, multiple reaction monitoring

## Abstract

The consumption of plant-based drinks is increasing, but they represent a product category normally with lower protein content as compared with bovine milk. Furthermore, the products are highly processed and, therefore, the proteins in this product category may carry a significant processing history. In the present study, a series of 17 freshly produced, commercially available plant-based drinks were benchmarked according to protein-quality parameters. The plant-based drinks represented different plant sources, as well as some mixed products, and were investigated relative to composition, aggregate sizes, presence of non-reducible proteins complexes, and level of processing-induced markers in the proteins. Processing-induced changes in the proteins were determined by a newly developed cocktail method, determining markers related to Maillard and dehydroalanine pathways, as well as intact lysine by triple quadrupole-multiple reaction monitoring-mass spectrometry. It was found that all drinks contained non-reducible protein complexes, but specifically, oat-based drinks represented the largest span contents of processing-induced markers within the proteins, which may relate to their inherent processing histories. Furthermore, it was shown that in products containing added sugar, Maillard reaction-related processing markers were increased over the dehydroalanine pathway.

## 1. Introduction

The consumer demand for plant-based drinks (PBDs) is increasing, especially among consumers from North America and Europe [1]. Initially, these products were mainly directed at segments of people with lactose intolerance or other nutritional restrictions like an allergy towards cow’s milk protein [2]. However, the increase in scientific evidence on environmental consequences of animal-based food systems has led to consumers being more concerned about their climate impact. With their milk-like appearance and consistency, PBDs act as a direct substitute to bovine milk for consumers wanting to live a more sustainable lifestyle [3]. Various plant sources are currently used for the typical PBDs, such as oat, rice, coconut, almond, pea, and soy, with almond and oat being the most consumed products in Europe and North America, whereas soy drink is more popular in Asian countries [4]. The nutritional content varies depending on plant source and formulation, but compared to cow’s milk, PBDs generally have lower protein contents, with soy being an exception [5]. Furthermore, most of the PBDs are made from a single plant-source, where the amino acid composition is less balanced since most plants are low in one or more essential amino acids, compared to the daily human requirement [6].

**Figure 1:**
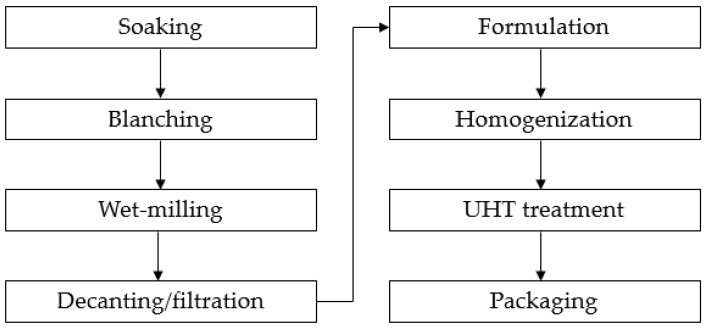
General flowchart of industrial-scale manufacturing process of PBDs [7,8,9].

PBDs can be produced with various processing set-ups, but a general outline of a modern industrial-scale process (Figure 1) would include initial soaking of the nuts or grains to soften the given plant source and make the homogenization process easier [7]. For oat-based drinks, it is necessary to add alpha-amylase during the soaking step to break down the starch and prevent gelatinization [8]. The next step would often include blanching or other heat treatment to eliminate microbial and enzymatic activities, e.g., the exogenous alpha-amylase in oat-based drinks or the indigenous enzyme lipoxygenase in soy, which causes oxidation and a beany-like flavor in soy-based drinks, if not inhibited [7]. Wet-milling produces a homogenous mix of the grinded plant-source in water, and this slurry is then filtrated or decanted to separate the coarse particles in the slurry. Food ingredients can help improve the stability, flavor and mouthfeel of the PBD through addition of, e.g., sugar, stabilizer, and vegetable oil, whereas improvement of the micronutrient content can be achieved by the addition of vitamins [7]. Homogenization of the slurry can be performed at various time points during processing; commonly, it is performed after the filtration and formulation step. The final step is heat treatment, usually at an ultra-high temperature (UHT), to improve suspension and secure food safety during storage [9]. 

During the processing steps, the plant proteins may undergo chemical changes and modifications that can have both short- and long-term consequences on product quality and nutritional value. Depending on product composition, such protein process-induced changes could be through the sugar-dependent Maillard reactions or the sugar-independent dehydroalanine (DHA) pathway [10]. These unwanted molecular protein-quality related changes can limit shelf life and lead to both immediate and storage-dependent changes in functionality and nutritional value [11].

The Maillard reactions are a cascade that can occur when proteins are heat treated in the presence of reducing sugars. It can be divided into two development stages; early and advanced. Early stages consist of the carbonyl group of a reducing sugar condensing with an amino group from, e.g., a lysine residue or from free amino terminals to form an unstable Schiff base [12], which normally rearranges into the more stable Amadori product. In the advanced stage, different advanced glycation end products (AGE) can be formed by degraded Amadori products and nucleophilic side chains of amino acids. Further reactions form brown-colored polymers, known as melanoidins. Furosine is an early stage marker for the Maillard reaction after acid hydrolysis, whereas N-ε-(carboxyethyl)lysine (CEL) and N-ε-(carboxymethyl)lysine (CML) are peptide- or protein-bound lysine modifications and markers of advanced-stage Maillard. CML can be formed through oxidation of Amadori products [11]. 

The DHA pathway is independent of reducing sugars, and results in formation of protein cross-linking induced by heat and/or alkaline conditions. Initially, DHA is formed through β-elimination of serine, cysteine or cystine residues, and the formed DHA can then react with either a lysine, cysteine or histidine residues to form the protein cross-links lysinoalanine (LAL), lanthionine (LAN), and histidinoalanine (HAL), respectively [11]. The Maillard reaction and DHA-mediated protein cross-linking are known to occur in long shelf-life dairy products, such as UHT milk [13], but less is known about their presence in PBDs. With furosine, CEL, and CML being markers of Maillard reaction and LAL, LAN and HAL as markers of DHA pathway, Nielsen et al., [10,11] reported a method for absolute quantification of processing-induced markers. The method is based on liquid chromatography coupled to a triple quadrupole mass spectrometer (LC-MS triple Q) method with multiple reaction monitoring (MRM), which enables absolute quantification of Maillard reaction markers simultaneously with DHA pathway markers.

The aim of this study was to investigate the protein quality in commercially available Danish PBDs made from oat, almond, pea, soy, barley, and hemp, from a protein quality and nutritional perspective. In this context, the protein quality is in relation to the interplay between plant source, PBD composition, protein content and particle size, aggregate formation, and level of processing-induced markers. This is a study on commercially available, but relatively freshly made, PBDs from Danish manufacturers.

It is hypothesized that quantity and nature of processing-induced markers in the protein parts of the PBDs will vary relative to compositional aspects (presence or absence of sugars and relative to individual protein composition) and the processing history carried by the products. It is further hypothesized that the Maillard reaction is favored over the DHA pathway in the presence of reducing sugar.

## 2. Materials and Methods

### 2.1. Samples of Plant-Based Drinks

A total number of 17 freshly produced PBDs of commercial availability in Danish supermarkets and from Danish manufacturers were included in the study. At the time of purchasing, the products were <4 weeks old and <2 weeks old for long shelf-life and short shelf-life products, respectively. In the present study, long shelf-life products refer to PBDs with a shelf life > 8 months, and short shelf-life products refer to PBDs with a shelf life < 7 weeks. After purchase, the drinks were aliquoted and frozen at −20 °C for further analyses. Table 1 (oat-based drinks) and Table 2 (mixed and non-oat PBDs) list the PBDs included in the study with their assigned sample name and nutritional contents. Ingredients, manufacturers and commercial product names are found in supplementary data.

### 2.2. Validation of Protein Contents in the PBDs

Protein concentrations were indicated on the packages, but were also validated by two different methods, Dumas and Bradford, in our laboratories to ascertain levels relative to the method. These values were used in connection to the determination and quantification of processing-induced protein modifications. The protein contents were initially determined based on total N by Dumas using Dumatherm (Gerhardt Analytical Systems, Königswinter, Germany). Samples of 100 µL PBD were added to DumaFoil containing DT SuperAdsorber (Gerhardt Analytical Systems, Königswinter, Germany). The samples were measured in technical duplicates and the standard was 0.5% tris(hydroxymethyl)aminomethane. The nitrogen-to-protein conversion factors for each plant source were as follows: oat = 5.83, almond = 5.18, pea = 5.3, soy = 5.71, barley = 5.83, and hemp seeds = 5.3 [14].

Protein concentration was further determined using the Bradford assay. Samples of PBDs were diluted in Milli-Q water based on declared protein content and 10 μL were added in technical triplicates to a 96-well microtiter plate. The Bio-Rad Protein assay dye (Coomassie Brilliant Blue, Bio-Rad Laboratories, Hercules, CA, USA) was diluted to 1:5 and 200 μL was added to each well. The plate was incubated for 10 min, and absorbance was measured with a Biotek Synergy 2 Plate Reader (Agilent Technologies, Santa Clara, CA, USA) at 595 mn. The standard curve was made using bovine serum albumin dissolved in Milli-Q water and pure Milli-Q water as the blank.

### 2.3. Sodium Dodecyl Sulphate Polyacrylamide Gel Electrophoresis (SDS-PAGE)

Samples of PBDs were electrophoresed, as described by Sheng et al. [15], with some modifications. The samples were diluted in Milli-Q water to a final concentration of 2 μg/μL protein, according to the declared protein content provided on the product packages, and mixed 1:1 with the Laemmli sample buffer (20 mM Tris, 2% SDS, 20% Glycerol, bromophenol blue, 20 mM dithioerythritol) under reducing conditions. The samples were heated to 90 °C for 2 min and centrifuged shortly, and 20 μL were loaded in wells of polyacrylamide gel Criterion Protein Gel (TGX Stain-Free, any kDa, Bio-Rad Laboratories, Hercules, CA, USA) and 5 μL of ProteinLadder (Bio-Rad Laboratories, Hercules, CA, USA) was applied as a marker. The gels were run at 200 V for 30–40 min on a Criterion Dodeca Cell (Bio-Rad Laboratories, Hercules, CA, USA) with a Laemmli running buffer (250 mM Tris-Base, 1.89 M Glycine, 10% SDS). The gels were fixed in 50% ethanol, 8% phosphoric acid for at least 2 h and then stained with Coomassie Blue (5% in Milli-Q, *w*/*v*) [15]. The gels were photographed with ChemiDoc XRS+ and processed with ImageLab software version 6.0 (Bio-Rad Laboratories, Hercules, CA, USA).

### 2.4. Particle Size

The average hydrodynamic particle size in PBDs were measured using the Zetasizer Lab (Malvern Panalytical, Malvern, UK). Samples of PBDs were diluted in a ratio of 1:50 in Milli-Q water and measured both before and after filtration (1.2 µm, Phoenex-GF/CA, Værløse, Denmark) with 1 mL transferred to a 1 cm × 1 cm cuvette. The scattered phase was PBD, whereas the continuous phase was Milli-Q water (refractive index 1.33). Samples were measured in Zetasizer 2000 and analyzed in the software ZS Xplorer version 2.0.0.98 (Malvern Panalytical, Malvern, UK).

### 2.5. Quantification of Levels of Processing-Induced Markers by MRM LC-MS Triple-Q

Sample preparation was conducted as described by Nielsen et al. [10]. Markers of processing-induced changes were analyzed in all included PBDs (Table 1 and Table 2). A volume comprising a total protein amount of 3 mg was drawn from each sample (81–1500 μL) and mixed 1:5 with ice-cold acetonitrile with 1% formic acid (FA) for the precipitation of proteins. The samples were then centrifuged at 4500× *g* for 20 min at 4 °C and the supernatant was removed. After this, 300 μL of 10 M HCl were added to the pellets, and the samples were incubated in glass vials for 24 h at 110 °C for acid hydrolysis. Then, 500 μL of LC-MS grade water was added. The hydrolyzed samples were vortexed, transferred to an Eppendorf tube with a glass pipette, put on ice for 30 min and centrifuged at 14,000× *g* for 15 min at 4 °C. The supernatant was collected and 6 M NaOH was added before filtering with 0.2 μm filter (Whatman, Buckinghamshire, UK) into LC-MS glass vials and a mixture of internal standards containing 0.1 μg/mL of CEL-d4, CML–d2, and Lysine-d4, 0.2 μg/mL of furosine-d4, and 1 μg/mL of Cystine-d4 was added. The samples were then injected into an LC Infinity 1260 system and analyzed on a 6460 triple-Q MS(Agilent Technologies, Waldbronn, Germany), which operated in MRM acquisition mode. Separation of the compounds released by acid hydrolysis was performed on an Intrada amino acid column (3 mm × 150 mm, 3 μm, Imtakt, Portland, OR, USA). The mass spectrometry method was conducted as previously described [11]. Quantification was based on the ratio of the analyte and internal standard with MassHunter Quantitative Analysis software version B.08.00 (Agilent Technologies, Santa Clara, CA, USA). The amount of each marker was expressed as mg/g protein from each product.

### 2.6. Statistical Analysis

Statistical analysis was conducted in the software Rstudio (version 2023.6.1., Boston, MA, USA). Differences in contents of processing-induced markers and lysine in the PBD, as well as particle size distribution, were determined by mean comparison with Tukey’s HSD post hoc test after a one-way analysis of variance (ANOVA) test on the data. Differences were considered significantly different at *p* ≤ 0.05. For protein content measurements, statistical differences between methods were determined in Microsoft Excel (2016) using a two-tailed paired *t*-test. 

## 3. Results and Discussion

### 3.1. Protein Contents and Compositions in the PBDs 

**Figure 2:**
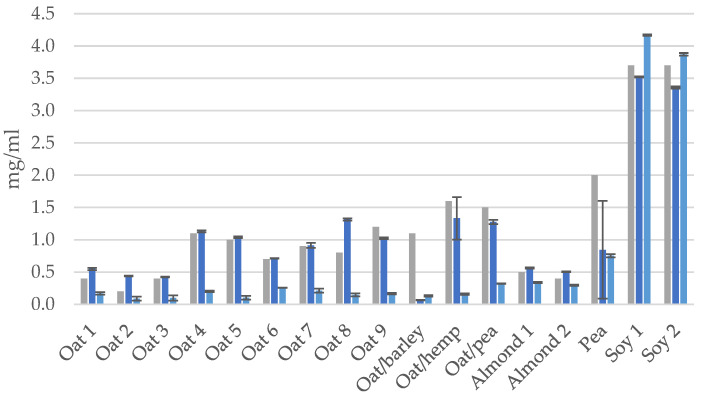
Protein content in PBD’s measured by Dumas or Bradford and compared with the declared value from manufacturers. Gray: declared, dark blue: Dumas, light blue: Bradford.

Protein contents in the PBDs were initially measured with Dumas and Bradford methods and compared with the declared value stated on the product packaging (Figure 2), in order to have a validated baseline for the protein contents in the PBDs and to be used in the quantification level of processing-induced markers in the proteins. For most PBDs, protein content that was measured by Bradford was significantly lower than both declared values and Dumas-based measurements (*p* ≤ 0.05). Exceptions include soy, which showed similar content across the measurements, whereas oat/barley or pea showed a lower protein content with both Dumas and Bradford, compared to the declared value. It was found that soy drinks contained more protein than any of the other PBDs and that almond- and oat-based drinks had the lowest protein content. 

The results suggest that the Dumas method or other similar methods (i.e., Kjeldahl method) are used in the industry to determine the protein content. Both the Dumas and Kjeldahl method determine the nitrogen content in samples, which is then converted to protein content using a provided conversion factor depending on the actual protein source. As some protein sources may contain non-protein nitrogen (NPN), methods based on N-determination may lead to an overestimation of the real protein content. NPN can come from nucleic acids, free amino acids or other N-containing compounds, which the nitrogen conversion factor does not take into account since the conversion factor is determined based on the average nitrogen content of the specific amino acids that make up the proteins in the protein source [16]. Pea- and soy-based drinks are sometimes made from protein concentrates, and not whole soy beans or peas; hence, there may be a loss of certain proteins during the protein purification process. If the loss is high-N proteins, the nitrogen conversion factor will underestimate the protein content, whereas if the products have added ingredients, i.e., vitamins or aromas, these may contribute to NPN, which can lead to an overestimation of protein content.

The Bradford protein assay relies on the proteins’ ability to bind to Coomassie dye under acidic conditions, resulting in a color change; hence, the proteins need to be in solution to be able to bind to the dye. Proteins that are aggregated or precipitated will not interact with the dye in the same way, and the assay may provide inaccurate or inconsistent results [17]. The majority of the PBD’s included in this study were UHT treated, in order to secure food safety and quality requirements, which in turn leads to protein denaturation and can cause aggregation and precipitation of the proteins, which means the proteins are no longer in a solution and unable to react with the dye. From the results in Figure 2, we can assume that the majority of the proteins are no longer in solutions since the Bradford measurements are lower than those for the Dumas results and the declared contents. The two soy drinks are an exception here, which may indicate less protein aggregation since they are still able to bind to the dye. Previous studies found that commercially available soy-based drinks had higher protein solubility compared to oat-based drinks [18].

**Figure 3:**
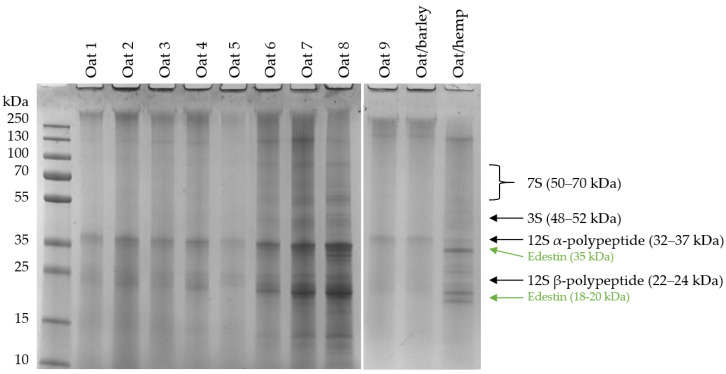
Tris-HCl (4–20%) gel electrophoresis of PBDs. 20 µg protein was added to each lane under reducing conditions and visualized with Coomassie Brilliant Blue. The protein ladder on the left indicates protein size, and on the right are identified proteins based on previous findings [19,20].

The protein compositions were investigated under reducing conditions by 1D SDS-PAGE for all PBD. Oat-based drinks are shown in Figure 3 and display two main protein bands eluting at positions equivalent to molecular masses of approximately 35 and 22 kDa. Based on identification from earlier studies, these two protein bands correspond to the molecular weights of the α-polypeptide, 32–37 kDa, and β-polypeptide, 22–24 kDa, from the major 12S storage protein found in oat [9,19]. Oat 6, 7 and 8 also contained subunits from the 7S protein, 50–70 kDa, and from 3S, 48–52 kDa [9,19]. The mixed oat/barley PBD did not seem to contain additional proteins compared with other oat drinks, hence, no protein from barley was found in the drink. Oat/hemp contained edestin subunits from hemp, 18–20 kDa and 35 kDa [20]. It is important to note that the same amount of protein was loaded onto the gel, however, there is still a visible variance in intensities of the protein bands, indicating that not all protein is able to enter the gel. From the top of the gel in Figure 3, it is evident that some aggregates are present in the well of the SDS-PAGE gel, even under reducing conditions. This means that the PBDs contained non-reducible aggregates, which, e.g., could come from processing-induced protein cross-linkages, leading to the proteins not being able to enter the gel due to their size.

**Figure 4:**
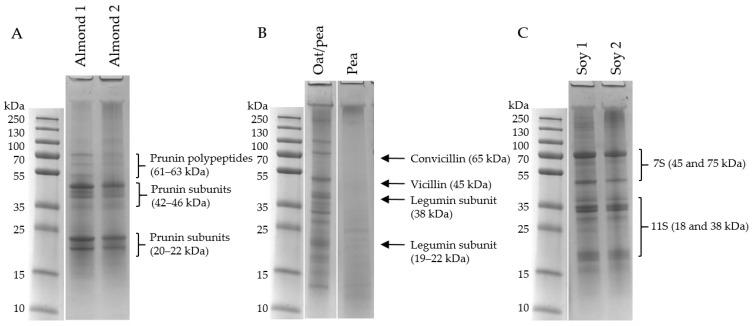
Tris-HCl (4–20%) gel electrophoresis of PBD made from almond (**A**), pea and in combination with oat (**B**) and soy (**C**). A total of 20 µg protein were added to each lane under reducing conditions and visualized with Coomassie brilliant blue. The protein ladder on the left indicates protein size, and on the right are identified proteins based on previous findings [21,22,23].

Figure 4 shows samples of almond (A), pea (B) and soy (C) drinks. The almond drinks contained prunin subunits, 61–63 kDa, and its acid subunit, 42–46 kDa, and basic subunit, 20–22 kDa [21]. Protein bands for the almond 1 drink were more clearly visible compared to almond 2, whereas we see more aggregated protein in the well of almond 2. The recipes for the two almond drinks were very similar (supplementary material), however, almond 2 was made with roasted almonds and had higher levels of sugar (Table 2) compared to almond 1. Therefore, it may be assumed that processing-induced changes, i.e., further heating from roasting almonds and more available sugars, can be the reason to why more aggregation is seen in almond 2 [22].

From Figure 4B, it was seen that oat/pea PBD had a relatively broad spectrum of proteins from pea, e.g., convicillin, 65 kDa, vicillin, 45 kDa, and subunits from legumin, 19–38 kDa [22], whereas the PBD based only on pea exhibited almost no proteins; hence, these two drinks were relatively diverse. The protein measurements of the pea drink had a high-standard deviation from Dumas, which could indicate that the drink was less homogenous and could explain why almost no protein bands were visible in the gel. Furthermore, there were also indications of protein aggregates visible in the top of the gel, meaning that the proteins present seem to be bound as non-reducible high-molecular weight complexes. 

Both soy drinks in Figure 4C were relatively similar and contained subunits from the 7S protein, 45 and 75 kDa and from the 11S protein, 18 and 38 kDa [22,23]. The electrophoretic patterns of both drinks were comparable, and both also displayed non-reducible complexes in the top of the well, however, the bands are a bit less pronounced in soy 2. Soy 1 is only made from water and soy beans, whereas soy 2 also contains rice, vanilla aroma and calcium; however, from the gels, these ingredients appear to not affect the protein profile.

### 3.2. Particle Size before and after Filtration of PBDs

The average hydrodynamic particle size, z-average, in the PBDs was measured before and after filtration (1.2 µm). Samples were found to be significantly different after filtration (*p* < 0.05, Table 3). In the unfiltered samples, the particle sizes ranged from 350–2000 nm across all PBDs with soy 1 and soy 2 having the smallest particle sizes of 443 nm and 377 nm, respectively, and oat 8 having the largest particle size of 1983 nm. Furthermore, oat 6, oat/pea and oat/hemp had average particle sizes above 1000 nm. Overall, the PBDs containing oat showed the highest heterogeneity in particle size compared with other plant sources. Oat/pea and pea were significantly different from each other, whereas the two almond and the two soy drinks showed no significant differences when containing the same plant source. Particle sizes of filtered samples were found to be between 200–360 nm with only oat 6, oat 8, and oat/pea having a particle size > 300 nm. A previous study found that similar particle sizes for soy drinks were around 400 nm and up to 1500 nm for oat drinks [18]. No clear relation was obvious between PBDs containing non-reducible protein complexes as detected by SDS-PAGE, and the particle sizes measured with the Zetasizer. 

### 3.3. Processing-Induced Markers

Levels of processing-induced markers into the plant proteins of the PBDs are shown in Table 4. Generally, relatively large variability in levels and type of modifications were observed between the different PBDs. Oat 1 and oat 5 contained significantly higher levels of the early Maillard reaction marker, furosine, compared to other PBD samples. Oat 1 also showed a higher content of the AGEs, CEL and CML, with CML being significantly higher levels than all other PBDs. Oat 1 also contained LAN at the significantly highest level but the level of lysine was the same for all drinks containing oat except the oat/pea drink. The two almond drinks differed significantly in their lysine levels and in furosine, which was below the limit of quantification in almond 2. In the Maillard reaction, Lysine can form Amadori products, which can rearrange into furosine or directly evolve into the advanced glycation end products, CEL and CML [11], which seemingly happened in almond 2. 

No significant difference was seen between the two soy drinks, despite soy 2 containing higher sugar levels than soy 1. From Table 2, it is evident that soy 1 contains 0.1 g/100 g and soy 2 contains 3.4 g/100 g sugar, which most likely come from soy 2 being a hybrid drink with added rice from where the sugar content originates (Supplementary Data). We see higher levels of both early and advanced stage Maillard reactions in soy 2 compared to soy 1, especially furosine, indicating that the sugar dependent pathway was favored, however, the differences were not significant. 

The oat-based drinks both displayed some of the highest and lowest levels of processing-induced markers, which could be linked to their specific ways of being produced, as well as the ingredients used. During processing of oat-based drinks, it is necessary to enzymatically break down the starch with a-amylase to avoid gelatinization after heating the drink, however, it is a possibility that the manufacturers use an oat syrup for their drinks, which has already been hydrolyzed and is therefore ready to use. It can be speculated that an ingredient like that may represent additional processing history and, thereby, potentially higher levels of processing-induced markers in the final product, for example, due to additional heat treatment of the syrup ingredient used. Comparing the level of processing-induced markers with the results from the SDS-PAGE gels, it was observed that that oat 6–8 exhibited more protein bands and, thereby, higher protein heterogeneity in the SDS-PAGE gels; however, levels of markers were not unique for these specific oat drinks, emphasizing the complexity of the drinks. 

Intact lysine content in the PBDs was furthermore determined by the LC-MS triple Q MRM method. This is interesting as lysine is a substrate of both the Maillard reaction and the DHA pathways. Furthermore, lysine is an important essential amino acid and lysine residues are targets for hydrolysis during digestion. The investigated lysine originated from protein, peptides and free lysine, as the method is based on complete hydrolysis of the protein into amino acids and their derivatives. The highest lysine contents were found in the two products containing soy and pea, whereas oat and almond products contained less lysine. In the spectrum of oat-based drinks, we see a correlation between the drinks containing the highest amounts of processing-induced markers having the lowest contents of unmodified lysine, which can indicate that the lysine was exerted on these protein cross-links.

It is noted that LAL, coming from reaction of DHA product (from serine, cysteine or cystine) with lysine, was below the detection limit in all PBDs, while the DHA pathway was represented by LAN, coming from DHA products reacting with cysteine. This indicates that, overall, the PBDs had more cysteine residues available for reaction with DHA than lysines, and that the reacting lysine residues were substrates to Maillard-related reactions and not DHA-related reactions in the PBDs. Nielsen et al. [10] found that from the DHA pathway, LAL was the most predominantly formed protein cross-link compared to LAN in semi-skimmed UHT milk, with levels four times higher than that of LAN. In the DHA pathway, cysteine reacts with DHA to form LAN, however, studies examining the amino acid contents of PBDs found lower or similar levels of cysteine as in milk, although this does not explain why LAN is the predominant product of the DHA pathway in PBDs [5]. 

## 4. Conclusions

In the present study, 17 Danish commercially available PBDs were benchmarked according to presence and sizes of protein aggregates and level of processing-induced markers within the proteins. It was found that the aggregate sizes varied from 350–2000 nm in the original drinks and that this was decreased by filtration to 200–260 nm. By SDS-PAGE, it was further shown that all PBD types contained non-reducible protein complexes, and that these could be due to protein–protein cross-links due to both Maillard reaction and DHA pathway-related changes in sugar containing PBDs, while in PBDs with only very low levels of sugar, the DHA pathway-related changes were prevalent. The DHA-related markers detected were related to LAN, thereby derived from the reaction of DHA with cysteine in the proteins, while the levels of LAL were below the detection limit. The level of non-modified lysine in the PBDs was lowest in those samples with the highest levels of processing-induced markers. Oat-based drinks exhibited the largest variation in levels of processing-induced markers, which may relate to variability in the carried processing histories of various products. 

## Figures and Tables

**Table 1:** Declared nutritional contents of oat-based drinks included in the study.

	Oat 1	Oat 2	Oat 3	Oat 4	Oat 5	Oat 6	Oat 7	Oat 8	Oat 9
Energy (kJ/kcal)	199/47	143/34	150/36	264/63	297/71	143/34	209/50	237/58	204/49
Protein (g/100 g)	0.4	0.2	0.4	1.1	1	0.7	0.9	0.8	1.2
Fat (g/100 g)	1.3	1.2	1.4	3.4	2.9	1.4	1.2	0.5	1.5
Saturated fat (g/100 g)	0.5	0.2	0.2	0.3	0.3	0.2	0.1	0.1	0.2
Carbohydrates (g/100 g)	8	5.6	5.3	6.7	9.6	4.3	8.5	12	7.1
Sugars (g/100 g)	3.2	3.1	3.6	1.6	4.1	0	3.7	2	3.3
Fibre (g/100 g)	1	N/A	0.5	0.7	1	0.5	N/A	N/A	N/A
Salt (g/100 g)	0.08	0.09	0.12	0.12	0.1	0.09	0.1	0.02	0.17
Calcium (m g/100 g)	N/A	120	120	120	120	N/A	120	N/A	N/A
Shelf life	9 m, RT	5 m, 5 °C	9 m, RT	9 m, RT	12 m, RT	9 m, RT	12 m, RT	9 m, RT	1–2 m, 5 °C
Heat treatment	UHT	UHT	UHT	UHT	UHT	UHT	UHT	N/A	UHT

Room temperature (RT). Ultra-high temperature (UHT). Not available N/A.

**Table 2:** Declared nutritional contents of other PBDs included in the study.

	Oat/Hemp	Oat/Barley	Oat/Pea	Almond 1	Almond 2	Pea	Soy 1	Soy 2
Energy (kJ/kcal)	220/52	206/49	241/59	87/21	89/21	142/34	147/35	217/52
Protein (g/100 g)	1.6	1.1	1.5	0.5	0.4	2	3.7	3.7
Fat (g/100 g)	1.4	1.4	2.3	1.2	0.9	2.1	2.1	2.2
Saturated fat (g/100 g)	0.3	0.1	0.3	0.1	0.1	0.2	0.4	0.4
Carbohydrates (g/100 g)	7.7	7.6	8	2	2.7	2	0.6	4
Sugars (g/100 g)	3.2	3.7	2.5	1.9	2.4	2	0.1	3.4
Fibre (g/100 g)	N/A	N/A	N/A	0.1	0.3	N/A	0.6	0.8
Salt (g/100 g)	0.13	0.12	0.3	0.1	0.1	0.2	0.04	0.16
Calcium (m g/100 g)	N/A	N/A	N/A	120	120	120	N/A	120
Shelf life	1–2 m, 5 °C	1–2 m, 5 °C	21 d, 5 °C	9 m, RT	9 m, RT	12 m, RT	9 m, RT	9 m, RT
Heat treatment	UHT	UHT	N/A	UHT	UHT	UHT	UHT	UHT

Room temperature (RT). Ultra-high temperature (UHT). Not available N/A.

**Table 3:** Particle sizes of PBDs measured with Zetasizer. All PBDs were diluted 1:50 in Milli-Q water and the measurement was conducted before and after filtration using 1.2 µm filters. Different superscript letters within a row indicate statistical significance (*p ≤* 0.05).

Sample	Z-Average [nm]			
	Before Filtration		After Filtration ^1^	
Oat 1	609.7 ^bcde^	±40.58	232.6 ^ab^	±4.05
Oat 2	742.2 ^defg^	±27.52	261.3 ^bcd^	±1.77
Oat 3	538.6 ^abcd^	±4.29	228.4 ^ab^	±4.04
Oat 4	802.3 ^efg^	±44.65	218.7 ^a^	±2.49
Oat 5	707.8 ^cdef^	±15.45	219.1 ^ab^	±1.68
Oat 6	1432.3 ^j^	±184.82	336.2 ^ef^	±31.33
Oat 7	933.3 ^gh^	±17.62	233.0 ^ab^	±4.61
Oat 8	1983.0 ^k^	±107.24	358.7 ^f^	±18.90
Oat 9	490.3 ^abd^	±14.59	235.5 ^abc^	±0.81
Oat/hemp	1070.0 ^hi^	±46.63	208.3 ^a^	±11.43
Oat/barley	720.8 ^defg^	±14.24	236.4 ^abc^	±6.62
Oat/pea	1201.3 ^i^	±53.32	300.3 ^de^	±20.09
Almond 1	604.7 ^bcde^	±16.87	275.9 ^cd^	±3.90
Almond 2	541.1 ^abcd^	±15.88	284.2 ^d^	±6.22
Pea	833.0 ^fg^	±62.70	224.0 ^ab^	±0.43
Soy 1	443.4 ^ab^	±7.66	285.5 ^d^	±4.04
Soy 2	377.1 ^a^	±4.95	225.5 ^ab^	±3.15

^1^ Filtration with 1.2 µm syringe filter.

**Table 4:** Levels of processing-induced markers and lysine in PBDs, as measured by MRM LC/MS triple Q. Processing-induced marker for early Maillard is furosine, and for advanced glycation end products, CEL and CML, and for DHA pathway, LAN. LAL was below the detection limit inall the PBDs. Different superscript letters within a row indicate statistical significance (*p ≤* 0.05). LOQ = limit of quantification.

Sample	Furosine		CEL		CML		LAN		Lys	
Oat 1	2.108 ^f^	±0.378	0.051 ^ef^	±0.014	0.440 ^e^	±0.090	0.772 ^d^	±0.190	22.305 ^abc^	±0.115
Oat 2	1.215 ^de^	±0.140	0.038 ^cde^	±0.006	0.270 ^d^	±0.030	0.513 ^cd^	±0.057	25.795 ^abc^	±0.585
Oat 3	0.181 ^abc^	±0.001	0.023 ^abcd^	±0.001	0.107 ^ab^	±0.001	0.211 ^abc^	±0.003	30.386 ^bc^	±1.617
Oat 4	0.496 ^abc^	±0.014	0.032 ^abcde^	±0.000	0.200 ^bcd^	±0.005	0.823 ^d^	±0.047	30.718 ^bc^	±1.932
Oat 5	1.271 ^e^	±0.049	0.047 ^ef^	±0.003	0.251 ^cd^	±0.019	0.282 ^abc^	±0.014	13.670 ^ab^	±0.073
Oat 6	0.004 ^a^	±0.000	0.042 ^de^	±0.003	0.553 ^e^	±0.029	0.349 ^bc^	±0.028	25.411 ^abc^	±0.165
Oat 7	0.232 ^abc^	±0.007	0.014 ^ab^	±0.000	0.107 ^ab^	±0.002	0.224 ^abc^	±0.008	34.786 ^c^	±0.090
Oat 8	0.135 ^abc^	±0.004	0.010 ^a^	±0.001	0.058 ^ab^	±0.002	0.174 ^ab^	±0.012	42.548 ^cd^	±5.358
Oat 9	0.708 ^cd^	±0.119	0.048 ^ef^	±0.006	0.270 ^d^	±0.048	0.785 ^d^	±0.153	37.380 ^c^	±5.183
Oat/hemp	0.403 ^abc^	±0.007	0.015 ^abc^	±0.003	0.108 ^abc^	±0.001	0.421 ^bc^	±0.031	20.088 ^abc^	±0.368
Oat/barley	0.612 ^bc^	±0.031	0.035 ^bcde^	±0.002	0.201 ^bcd^	±0.007	0.263 ^abc^	±0.019	30.865 ^bc^	±3.215
Oat/pea	0.101 ^abc^	±0.002	0.069 ^fg^	±0.001	0.077 ^ab^	±0.000	0.159 ^ab^	±0.015	65.461 ^de^	±5.578
Almond 1	0.039 ^a^	±0.000	0.083 ^gh^	±0.001	0.062 ^ab^	±0.001	0.132 ^ab^	±0.009	33.080 ^bc^	±2.400
Almond 2	<LOQ		0.102 ^h^	±0.005	0.039 ^ab^	±0.002	0.112 ^ab^	±0.005	5.667 ^a^	±0.547
Pea	0.209 ^abc^	±0.008	0.050 ^ef^	±0.000	0.172 ^bcd^	±0.001	0.035 ^a^	±0.001	63.941 ^e^	±0.736
Soy 1	0.008 ^a^	±0.000	0.053 ^ef^	±0.003	0.036 ^a^	±0.003	0.225 ^abc^	±0.011	63.386 ^de^	±6.030
Soy 2	0.255 ^abc^	±0.012	0.065 ^fg^	±0.003	0.129 ^abcd^	±0.007	0.290 ^abc^	±0.013	72.706 ^e^	±13.150

## Data Availability

Data is contained within the article and Supplementary Material.

## Supplementary Materials

The following supporting information can be downloaded at: https://www.mdpi.com/article/10.3390/foods12173282/s1, Table S1. Declared contents of PBDsc, their name and manufacture company.
