# Supplementary material for: Processing-Induced Markers in Proteins of Commercial Plant-Based Drinks in Relation to Compositional Aspects
Source: Foods. 2023 Sep 1;12(17):3282. doi: 10.3390/foods12173282 (PMC10487255; doi:10.3390/foods12173282)
Supplement: Supplementary file 1 [file foods-12-03282-s001.zip › foods-2559196-supplementary.pdf]

Table S1. Declared contents of PBDsc, their name and manufacture company. The commercial names are in some cases translated to English.

Sample Name	Content	Commercial name	Company
Oat 1	Water, 16% oat, sunflower oil, inulin, seasalt	Oat	Naturli' Foods A/S
Oat 2	Water, oat (8%), sunflower oil, algae lithothamnium calcareum, sasalt, gellan and guar gum	Oat drink	Naturli' Foods A/S
Oat 3	Water, oat (9.5%), sunflower oil, calciumcarbonat, salt, gellangum, vitamin D, B2, B12	Oat Plus	Naturli' Foods A/S
Oat 4	Water, oat (15%), rapeseed oil, tricalcium phosphate, calciumcarbonat, seasalt, vitamins D, B2, B12	Oats for Coffee	Naturli' Foods A/S
Oat 5	Water, oat (13%), rapeseed oil, tricalcium phosphate, seasalt, gellan gum, vitamin D, B2, B12	Oat Barista	Naturli' Foods A/S
Oat 6	Water, gluten-free oat (10%), sunflower oil, vanilla aroma, salt, inulin, aroma, gellan gum	Oat Zero	Naturli' Foods A/S
Oat 7	Oat base (water, oat 10%), rapeseed oil, calcium carbinate, calcium phosphate, salt, vitamins (D3, B2, B12)	Oat	Dryk
Oat 8	Water, oat (15%)	Plant 0.5	Mill-Life
Oat 9	Water, oat (15%), rapeseed oil, salt	Oat	Jörd, Arla Foods
Oat/hemp	Water, 15% oat, hemp paste (0.75%), salt	Oat and Hemp	Jörd, Arla Foods
Oat/barley	Water, oat (12.5%), barley malt extract (2.3%) rapeseed oil, salt	Oat and Barley	Jörd, Arla Foods
Oat/pea	Water, 13% oat, 1% pea protein, 1% rapeseed oil, 1% salt	Oats and More	Thise
Almond 1	Water, 2% almond, cane sugar, lithothamnium calcareum, seasalt, gellan gum, guar gum	Almond Drink	Naturli' Foods A/S
Almond 2	Water, cane sugar, 2% roasted almond, lithothamnium calcareum, seasalt, gellan gum, guar gum	Roasted Almond	Naturli' Foods A/S
Pea	Water, 2.5% pea protein, agave sirup, rapeseed olie, dikaliumphosphat, calcium carbonat, calcium phosphate, oat olie, salt, vitamins (D2, B2, B12)	Pea	Dryk
Soy 1	Water, 10% soy beans	Soy Drink	Naturli' Foods A/S
Soy 2	Water, 10% soy beans, rice, lithothamnium calcareum, vanila aroma, seasalt	Soy with Calcium and vanilla	Naturli' Foods A/S
